# Older Adult’s Marital Status, Support Exchanges and Psychological Well-being in Everyday Life

**DOI:** 10.1093/geroni/igab046.3325

**Published:** 2021-12-17

**Authors:** Yee To Ng, Karen Fingerman

**Affiliations:** 1 University of Texas at Austin, Austin, Texas, United States; 2 The University of Texas at Austin, Austin, Texas, United States

## Abstract

Social support exchanges are an integral part of older adults’ well-being. Yet, we know little about how older adults' marital status may influence their support exchanges with different social partners in everyday life, and whether the effect of support exchanges on daily well-being vary by marital status. Adults aged 65+ (N = 278) completed an initial interview about their background and close social networks; then, participants reported whether they provided or received support from their close social partners and rated their psychological well-being for 5 to 6 days. Multilevel logistic models revealed that married older adults were more likely to provide or receive daily support from their close partners than widowed or divorced older adults. However, with respect to specific non-spousal ties, married older adults were less likely to provide support to siblings, friends or others (acquaintances, neighbors) compared to divorced older adults. Although married older adults were more likely to receive support from children than divorced older adults, they were less likely to receive support from siblings and friends compared to widowed or divorced older adults. Furthermore, receiving support from other familial ties (grandchild, other relatives) was associated with reduced daily well-being for widowed older adults whereas married older adults were able to maintain their daily well-being in such situation. Findings highlight the central role siblings and friends play in unmarried older adults' daily support networks and suggest that receiving support could have differential impact on daily well-being depending on older adults’ marital status.

